# Localization of muscarinic acetylcholine receptor-dependent rhythm-generating modules in the *Drosophila* larval locomotor network

**DOI:** 10.1152/jn.00106.2021

**Published:** 2022-03-16

**Authors:** Julius Jonaitis, James MacLeod, Stefan R. Pulver

**Affiliations:** School of Psychology and Neuroscience, University of St Andrews, St Andrews, United Kingdom

**Keywords:** central pattern generators, fictive motor patterns, fruit fly, muscarinic receptors, soft-bodied locomotion

## Abstract

Mechanisms of rhythm generation have been extensively studied in motor systems that control locomotion over terrain in limbed animals; however, much less is known about rhythm generation in soft-bodied terrestrial animals. Here we explored how muscarinic acetylcholine receptor (mAChR)-modulated rhythm-generating networks are distributed in the central nervous system (CNS) of soft-bodied *Drosophila* larvae. We measured fictive motor patterns in isolated CNS preparations, using a combination of Ca^2+^ imaging and electrophysiology while manipulating mAChR signaling pharmacologically. Bath application of the mAChR agonist oxotremorine potentiated bilaterally asymmetric activity in anterior thoracic regions and promoted bursting in posterior abdominal regions. Application of the mAChR antagonist scopolamine suppressed rhythm generation in these regions and blocked the effects of oxotremorine. Oxotremorine triggered fictive forward crawling in preparations without brain lobes. Oxotremorine also potentiated rhythmic activity in isolated posterior abdominal CNS segments as well as isolated anterior brain and thoracic regions, but it did not induce rhythmic activity in isolated anterior abdominal segments. Bath application of scopolamine to reduced preparations lowered baseline Ca^2+^ levels and abolished rhythmic activity. Overall, these results suggest that mAChR signaling plays a role in enabling rhythm generation at multiple sites in the larval CNS. This work furthers our understanding of motor control in soft-bodied locomotion and provides a foundation for study of rhythm-generating networks in an emerging genetically tractable locomotor system.

**NEW & NOTEWORTHY** Using a combination of pharmacology, electrophysiology, and Ca^2+^ imaging, we find that signaling through mACh receptors plays a critical role in rhythmogenesis in different regions of the *Drosophila* larval CNS. mAChR-dependent rhythm generators reside in distal regions of the larval CNS and provide functional substrates for central pattern-generating networks (CPGs) underlying headsweep behavior and forward locomotion. This provides new insights into locomotor CPG operation in soft-bodied animals that navigate over terrain.

## Graphical abstract

**Figure:**
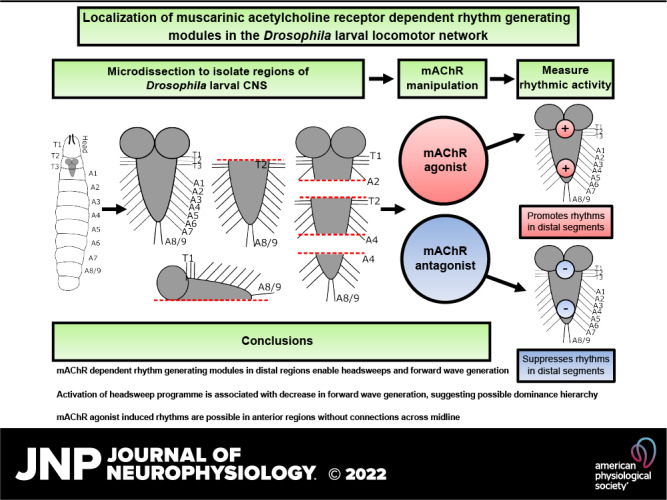

## INTRODUCTION

Understanding how and where rhythms are generated in motor systems is fundamental to understanding how animals move. The operation and location of central pattern-generating (CPG) networks controlling terrestrial locomotion has been extensively studied in limbed animals such as mice (1, 2), cats (3, 4), turtles (5), crustaceans (6, 7), and insects (8–10). In contrast, much less is known about CPG networks in soft-bodied terrestrial animals. Soft-bodied crawlers are intriguing from a motor control perspective; their fluid- and air-filled semicompressible bodies have high degrees of freedom of movement and present a unique set of motor control challenges that are different from those faced by limbed animals (11–13).

Work in leeches, caterpillars, and nematodes has provided important initial insights into the organization of CPG networks controlling soft-bodied crawling. In both leeches and caterpillars, there is strong evidence that crawling CPGs are organized as chains of coupled segmental oscillators (14, 15). In contrast, nematode crawling CPG circuits appear to be more continuous, with circuits spread across segmental boundaries (16–18). In all three systems, neuromodulators play critical roles in reconfiguring locomotor circuits to select one type of motor program over another (14, 15, 19).

The *Drosophila* larval locomotor system presents a unique opportunity to further study the neural control of locomotion in a genetically tractable soft-bodied animal. Recent work in this system has focused on characterizing the functional roles of identified interneurons and on uncovering functional circuit motifs based on electron microscopy (EM) reconstructions and selective activation and inhibition of interneuron subtypes in the central nervous system (CNS) (20–26). Previous work has revealed that the isolated larval CNS generates fictive motor programs for forward locomotion, backward locomotion, and headsweeps (27) and has provided initial insights into the location of CPG networks (28–31). These fictive motor programs are slower and more irregular than those observed in intact animals but retain qualitative and quantitative features observed in intact animals (27). Computational studies have modeled the larval ventral nerve cord (VNC) as comprised of oscillators in each hemisegment (32) or as recurrent networks (33); however, the precise location and neuromodulatory requirements for rhythm generation in this system have remained unclear.

Signaling through metabotropic cholinergic muscarinic receptors (mAChRs) is critical for rhythm generation in a variety of insect locomotor networks (14, 34–37). The underlying genes and pharmacological profiles of three separate *Drosophila* mAChR variants have been well characterized, with two variants (A and B types) showing pharmacological profiles similar to orthologous receptors in vertebrates (38, 39). Recent work has also characterized the behavioral and physiological effects of inhibition of expression of mAChRs in defined neuronal populations with RNA interference (RNAi) (40). Although powerful, these genetic manipulations operate over a timescale of days, leaving open the possibility of compensatory changes over developmental time. To date, no studies have directly explored the role(s) that acute manipulation of mAChR signaling plays in modulating CPG activity in defined regions of the larval locomotor system.

Here we use a combination of physiology, microsurgery, and pharmacology to explore the basic organization of muscarinic-dependent rhythm generation in the larval VNC. We show how mAChR-modulated rhythm-generating modules are distributed across the *Drosophila* larval VNC and provide evidence for the presence of multiple muscarinic-dependent rhythm-generating regions. This work provides a foundation for unraveling the cellular mechanisms of rhythm generation in the *Drosophila* larval locomotor system.

## MATERIALS AND METHODS

### Animal Rearing and Genetic Constructs

Flies were grown on standard cornmeal-based fly food. Animals were maintained in temperature-controlled incubators at 21–22°C with ∼55–60% humidity. The GAL4-UAS system (63) was used to drive expression of the Ca^2+^ indicator GCaMP6s (41) in all neurons. We used 57C10-GAL4 inserted in the attP2 landing site for all experiments. This GAL4 incorporates the promoter fragment for a common presynaptic protein (synaptobrevin) and has been used in previous panneuronal imaging studies (27, 42). We used a construct that has 57C10-GAL4 combined with UAS-GCaMP6s inserted in the attP40 landing site.

### Dissection

**Figure 1.**
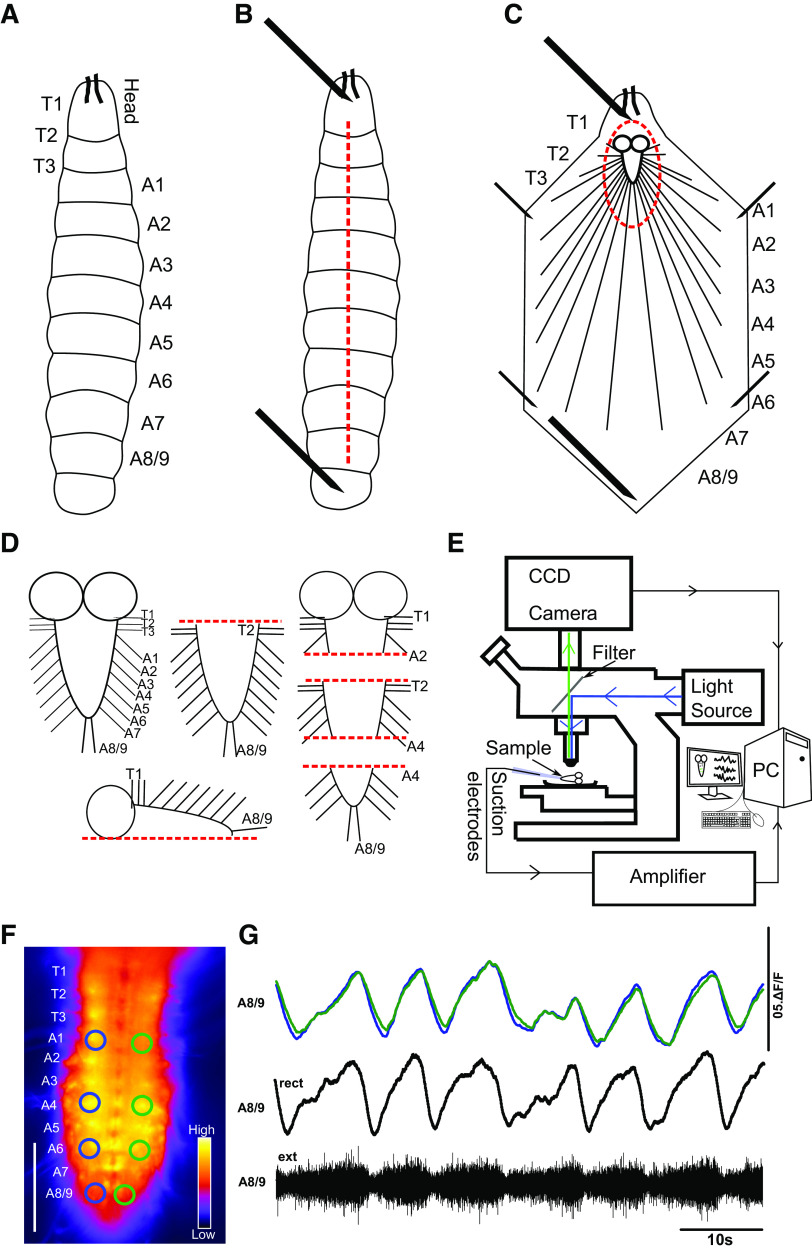
*Drosophila* larval preparation for recording fictive locomotor patterns. *A*: schematic of the body of a 3rd instar *Drosophila* larva. T1–T3 denote larval thoracic segments; A1–A8/A9 denote abdominal segments. *B*: schematic showing first steps of dissection. Head and tail of the larva are secured with insect pins. Dashed red line indicates where dorsal incision is made with microdissection scissors. *C*: schematic of larval body filleted with central nervous system (CNS) exposed and ready for removal. Dashed lines indicate where cuts are made to remove the CNS. *D*: schematic of isolated CNS and cuts made to isolate different CNS regions. Clockwise from *top left*: intact CNS; brain lobes removed; abdominal segments removed; brain lobes and distal regions removed; brain lobes and anterior regions removed; 1 side of CNS removed. *E*: experimental setup for simultaneous Ca^2+^ imaging and electrophysiological recordings. *F*: single frame showing raw fluorescence from an experiment in which Ca^2+^ activity is imaged panneuronally. Region of interest (ROI) size and placement are shown on the right (green) and left (blue) hemisegments, along with segment designations (on *left* of image). Differences in fluorescence intensity are indicated by color lookup table bar at *bottom right*. Scale bar, 100 μm. *G*: % change in fluorescence (ΔF/F) within A8/9 ROIs shown in *F* (scale bar shows 50% ΔF/F). Simultaneous extracellular nerve root recording from A8/9 confirms that Ca^2+^ signals from ROIs reflect motor output from hemisegments. Extracellular recordings (ext) are rectified and low pass filtered to visualize bursting (rect).

Feeding 3rd instar larvae were used for all experiments. In each experiment, single larvae were placed in an acrylic recording chamber lined with a thin layer (1 mm) of Sylgard 184 silicone elastomer (Sigma-Aldrich, Irvine, UK). Each larva was placed in the center of the chamber dorsal side up and pinned to the substrate by placing one insect pin in the mouth hook region and one pin in the most posterior abdominal segments (Fig. 1*A*). A dorsal incision was then made the length of the body with microdissection scissors (Fig. 1*B*). Four fine tungsten wire pins (0.025 mm; Alfa Aesar, Fisher Scientific) were then used to pin the larval body wall flat onto the surface of the dish, and internal organs of the larvae were then carefully removed, leaving the CNS attached to the body wall (Fig. 1*C*). The CNS, including the brain lobes and the ventral nerve cord (VNC), was then carefully cut out and placed away from the larval body carcass. The isolated CNS was pinned out dorsal side up by placing two to four fine tungsten wire pins around the preparation (California Fine Wire, Grover Beach, CA). Care was taken not to damage the brain lobes or VNC. In some experiments, different regions of the CNS were ablated (Fig. 1*D*). Ablations were made with microdissecting scissors and/or sharpened hypodermic needles (Sigma-Aldrich, Irvine, UK). Before recordings from ablated preparations proceeded, remaining nerve roots were counted to determine which segments were still intact and which had been removed. Preparations that showed clear evidence of structural damage away from the immediate region where the cut was made were excluded from analysis. During all dissections animals were submerged in physiological saline containing (in mM) 135 NaCl, 5 KCl, 2 CaCl_2_, 4 MgCl_2_, 5 TES, and 36 sucrose, pH 7.15. During dissection the saline was exchanged two or three times to remove debris.

### Electrophysiology

In a subset of experiments, extracellular suction electrode recordings from motor nerve roots projecting from the VNC were performed to record fictive motor patterns. Borosilicate glass capillary tubes (Kwik-Fil 1B150F-4; World Precision Instruments, Hitchin, UK) were pulled with a model P90 micropipette puller (Sutter Instruments, Hitchin, UK), and the tips were broken to match the diameter of a single nerve root. Three electrodes were then maneuvered toward the isolated CNS with a MP-285 (Sutter Instruments) mechanical micromanipulator and two manual manipulators (DT3-100 XYZ and DT3-130 XYZ; Siskiyou, Grants Pass, OR). Single nerve roots of interest were then drawn into the suction electrodes. Typically, one electrode was placed on a posterior nerve root (A7 or A8/9) and two placed on contralateral anterior nerve roots (T3 or A1) Electrophysiological signals were amplified with a model 1700 extracellular amplifier (A-M Systems, Sequim, WA), digitized with a model NI-USB-6229 data acquisition board (National Instruments, London, UK), and recorded with WinFluor V4.0.3 (University of Strathclyde, Glasgow, UK). Recordings were then analyzed offline with Spike2 version 7.08 b (Cambridge Electronic Design Limited, Milton, UK) or DataView (Bill Heitler, University of St Andrews, UK).

### Ca^2+^ Imaging in the Isolated CNS

Isolated CNS preparations expressing GCaMP6s panneuronally were imaged with a modified BX51 epifluorescence microscope (Olympus, Japan). An OptoLED (Cairn Instruments, Faversham, UK) was used to generate blue light (470 nm), which was directed to the preparation with a dichroic mirror in a custom-built housing (Cairn Instruments, Faversham, UK). Emitted light was filtered with a green fluorescent protein (GFP) emission filter. Images were captured with an iXon3 EMCCD camera (Andor Instruments, Belfast, UK) (Fig. 1*E*). Preparations were recorded for 25–30 min (depending on the experimental protocol) with a N20X-PFH ×20 water immersion objective (Olympus, Japan). Images were typically acquired at 9.94 frames/s with a 100-ms exposure time with WinFluor. Changes in fluorescence values were extracted from regions of interest (ROIs) in thoracic (T2–3) and abdominal (A2, 4, and 6) segments (Fig. 1*F*) with Fiji or ImageJ (43, 44), available online (https://imagej.net/). Extracted ROI fluorescence signals were visualized as percent change in fluorescence from average values (ΔF/F) with custom scripts written in Python (Python Software Foundation, www.python.org).

### Application of Pharmacological Agents

A custom-built superfusion system incorporating a U120 peristaltic pump (Watson-Marlow Pumps, Falmouth, UK) was used to superfuse physiological saline over preparations at a rate of 0.1 mL/s. Drugs were bath applied and washed out by switching between different gravity-fed inflows. For most experiments, control periods typically lasted 300–600 s, drug applications typically lasted 400 s, and wash periods lasted 400–800 s. In some experiments involving ablation of CNS regions, drug application periods were sometimes shortened to 200 s. It typically took ∼45–60 s for full bath exchange after switching feeds. Analysis windows were adjusted to account for time of bath exchange. Minimum time of analysis window in any experiment was 200 s. In ablation experiments, we typically waited at least 8–10 min after each cut before conducting experiments to reduce the chances of artifacts due to injury firing. The muscarinic agonist oxotremorine (Sigma-Aldrich, Irvine, UK) was applied at concentrations ranging from 10^−4^ to 10^−6^ M. The muscarinic antagonist scopolamine (Sigma-Aldrich, Irvine, UK) was applied at concentrations ranging from 3 × 10^−4^ to 3 × 10^−7^ M.

### Waveform Analysis, Statistics, and Figure Making

Event detection and waveform analysis were carried out with custom scripts in Spike2 and DataView. Criteria for determining type of activity pattern followed conventions established in previous work (27). ROI signals were analyzed from both sides of any given segment. ROI signals from one hemisegment were subtracted from the corresponding hemisegment across the midline to visualize bilateral asymmetries. Fictive headsweeps were defined as events with synchronous activity peaks in at least two adjacent segments on one side of the VNC while also showing at least a 4% difference in ΔF/F between left and right ROIs in both segments. Events per minute during analysis windows was calculated for each type of activity pattern (waves, headsweeps, posterior bursts). Statistical tests were carried out in GraphPad Prism 5 (GraphPad, San Diego, CA). One-way repeated-measures ANOVA with Bonferroni multiple comparison post hoc tests were used to make statistical comparisons. When data sets failed the Shapiro–Wilk normality test (*P* < 0.05), nonparametric Friedman with Dunn’s post hoc multiple comparison tests were used. Sample sizes represent data from individual animals. Figures were made in GraphPad Prism 5 or 8 and finalized in Inkscape (available free online at https://inkscape.org/).

## RESULTS

**Figure 2.**
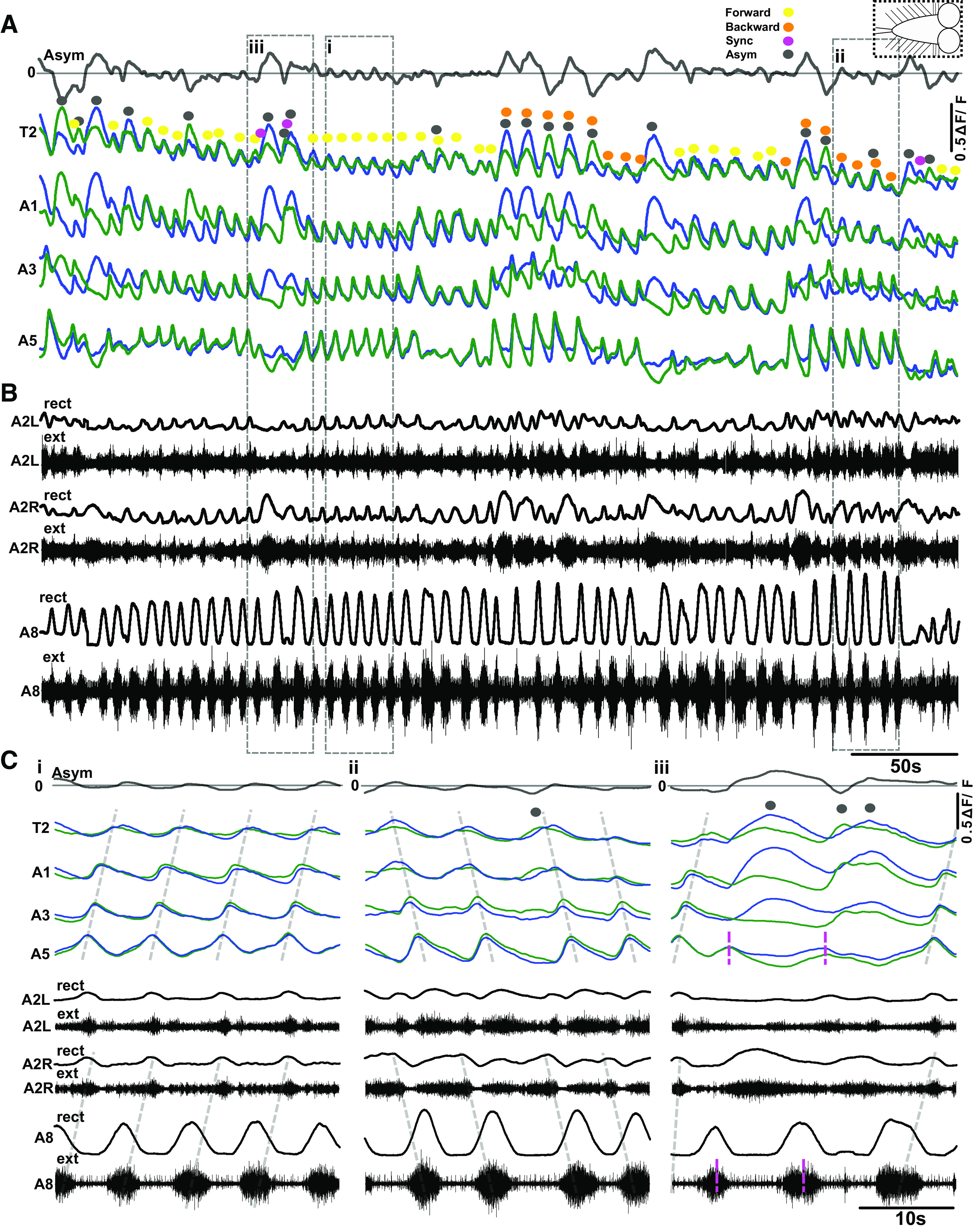
Spontaneous activity in the isolated larval central nervous system (CNS). *A*: % change in fluorescence (ΔF/F) extracted from regions of interest (ROIs) placed on different hemisegments on left (blue) and right (green) sides of the ventral nerve cord (VNC). Bilateral asymmetry (Asym) in Ca^2+^ intensity (dark gray trace, *top*) indicated between T2 left and right sides. Peaks above 0 baseline (light gray line, *top*) indicate higher Ca^2+^ intensity on the left side and below 0 indicate higher Ca^2+^ intensity on the right side of the VNC. Vertical scale bar indicates 50% ΔF/F. Dashed box at *top right* shows schematic of type of CNS preparation used in experiment. Colored dots indicate different types of motor activity (see color code at *top right*). *B*: simultaneous extracellular (ext) suction electrode recordings from 3 nerve roots (black traces), abdominal segment left and right sides (A2L and A2R), and single nerve root recording from abdominal segment 8 (A8). Extracellular nerve signals recorded from each electrode were rectified and smoothed by filtering with a moving average filter with a time constant of 0.9 s (rect). *C*: *i*: Expanded view of both Ca^2+^ signals and suction electrode recordings showing forward waves (dashed gray line). *ii*: Examples of backward waves. *iii*: Examples of bilateral asymmetries (black dots over events). Posterior bursts that did not propagate into forward waves are shown with magenta dashed lines.

To establish the fundamental features of larval locomotor rhythms, we recorded fictive locomotor activity using Ca^2+^ imaging combined with electrophysiological recordings from nerve roots in 3rd instar larval VNCs (Fig. 1, *F* and *G* and Fig. 2). Imaging Ca^2+^ panneuronally with a highly sensitive GCaMP variant (6 s) while also recording from multiple nerve roots maximized chances of detecting rhythmic activity in both interneuron and motor neuron populations (45).

**Figure 3.**
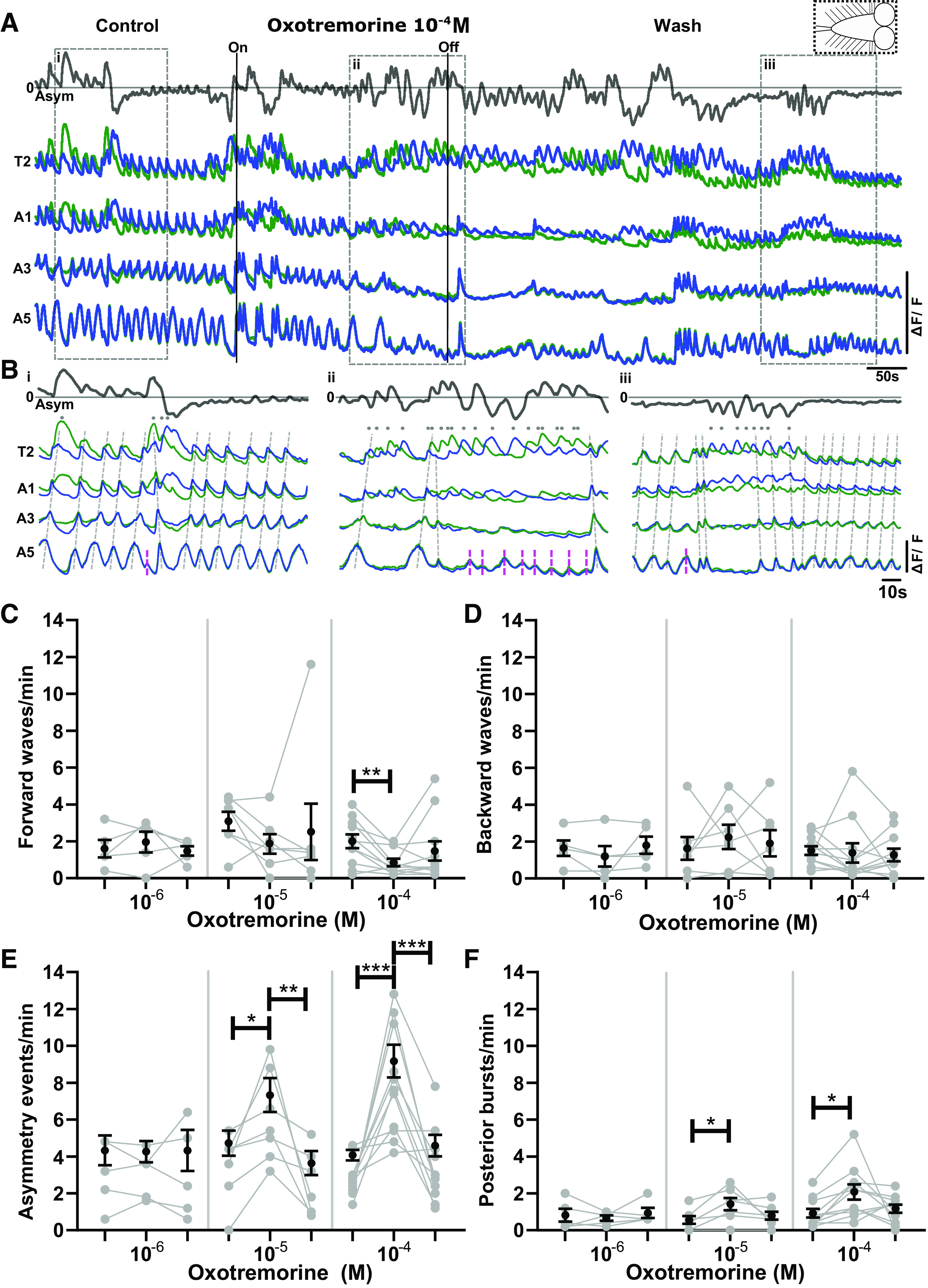
The muscarinic agonist oxotremorine increases frequency of bilaterally asymmetric activity in anterior regions and bilaterally symmetric bursts in posterior regions. *A*: % change in fluorescence (ΔF/F) extracted from regions of interest (ROIs) placed on different segments on left (blue) and right (green) sides of the ventral nerve cord (VNC). Bilateral asymmetry (Asym) in Ca^2+^ intensity (dark gray trace, *top*) presented as subtraction of Ca^2+^ signals on left from right sides. Dashed box at *top right* shows schematic of type of central nervous system (CNS) preparation used in experiment. Scale bars indicate 50% ΔF/F. *B*: *i*: Expanded view of Ca^2+^ signals during control period. *ii*: Expanded view of Ca^2+^ signals during oxotremorine 10^−4^ M application. *iii*: Expanded view of Ca^2+^ signals in wash period. *C–F*: events per minute of different fictive motor patterns produced in control, drug, and wash periods (from *left* to *right*) during application of different concentrations (M) of oxotremorine. Mean ± SE shown in black; data from each individual preparation shown in gray. Significant differences between different conditions of the experiment [1-way repeated-measures ANOVA with Bonferroni multiple comparison post hoc test; when data sets failed the Shapiro–Wilk normality test (*P* < 0.05), nonparametric Friedman with Dunn’s post hoc multiple comparison was used]: *P < 0.05, **P < 0.005, ***P < 0.0001. *C* and *D*: 10^−4^ M, 10^−6^ M. *E*: 10^−6^ M. *F*: 10^−5^ M. Sample sizes: *n* = 5 for 10^−6^ M, *n* = 7 for 10^−5^ M, and *n* = 11 for 10^−4^ M; event numbers range from 4 to 113.

Reading out Ca^2+^ signals in each hemisegment of the VNC provided a summed measure of all activity within that hemisegment; however, this approach did not enable us to explicitly distinguish between interneuron and motor neuron activity. Overlaying signals in corresponding segments across the midline allowed visualization of bilaterally symmetric and asymmetric events (Fig. 2 and Fig. 3, *A* and *B*, green and blue traces). Subtracting signals from corresponding ROIs across the midline allowed visualization and detection of bilaterally asymmetric activity patterns (Fig. 2*A*, Fig. 3*A*, gray trace at *top*). Simultaneous extracellular recordings were performed from multiple nerve roots in multiple types of preparations to confirm that Ca^2+^ signals in the panneuronal expression pattern in the larval VNC corresponded to motor activity (Fig. 1, *F* and *G*, and Fig. 2).

**Figure 4.**
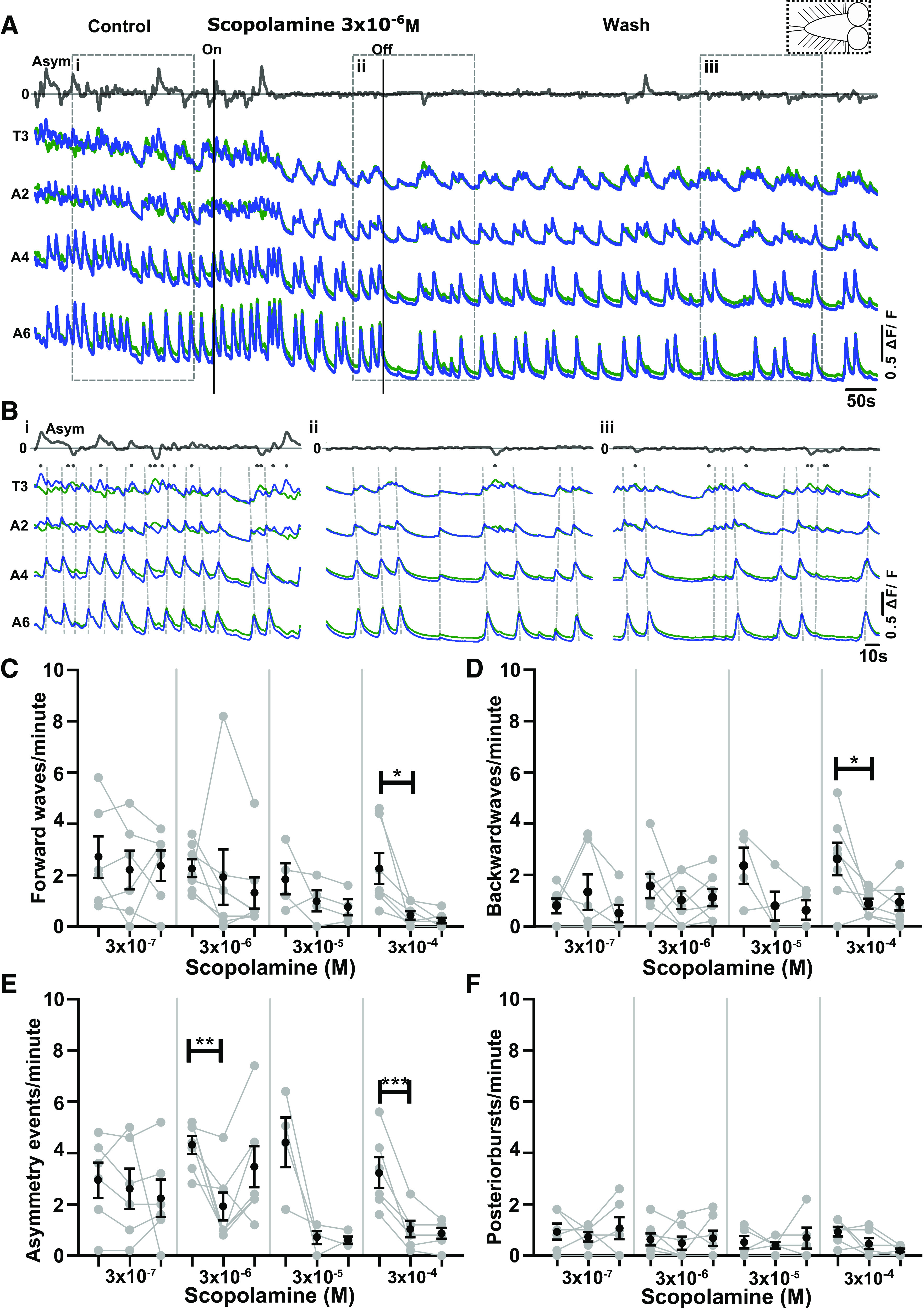
The muscarinic antagonist scopolamine inhibits fictive motor patterns in the isolated central nervous system (CNS). *A*: Ca^2+^ signals in an isolated CNS preparation during application of 3 × 10^−6^ M scopolamine. Gray trace at *top* represents subtraction of left and right side signals in T3. Dashed box at *top right* shows schematic of CNS sections present in experiment. ΔF/F, % change in fluorescence *B*: *i*: Expanded view of Ca^2+^ signals during control period. *ii*: Expanded view of Ca^2+^ signals during scopolamine application. *iii*: Expanded view of Ca^2+^ signals in wash period. *C–F*: events per minute of different fictive motor patterns produced before, during, and after (*left* to *right*) application of different concentrations of scopolamine. Mean ± SE shown in black; data from each individual preparation shown in gray. Significant differences among groups: *P < 0.05, **P < 0.005, ***P < 0.0001; 1-way repeated-measures ANOVA with Bonferroni post hoc test. For data sets that failed the Shapiro–Wilk normality test, nonparametric Friedman tests with Dunn’s post hoc multiple comparison were used. *C*: 3 × 10^−7^ M, 3 × 10^−6^ M, 3 × 10^−5^ M. *D*: 3 × 10^−7^ M, 3 × 10^−5^ M. *E* and *F*: all concentrations. Sample sizes: *n* = 6 for 3 × 10^−7^ M, *n* = 7 for 3 × 10^−6^ M, *n* = 4 for 3 × 10^−5^ M, *n* = 7 for 3 × 10^−4^ M; event numbers range from 4 to 85.

**Figure 5.**
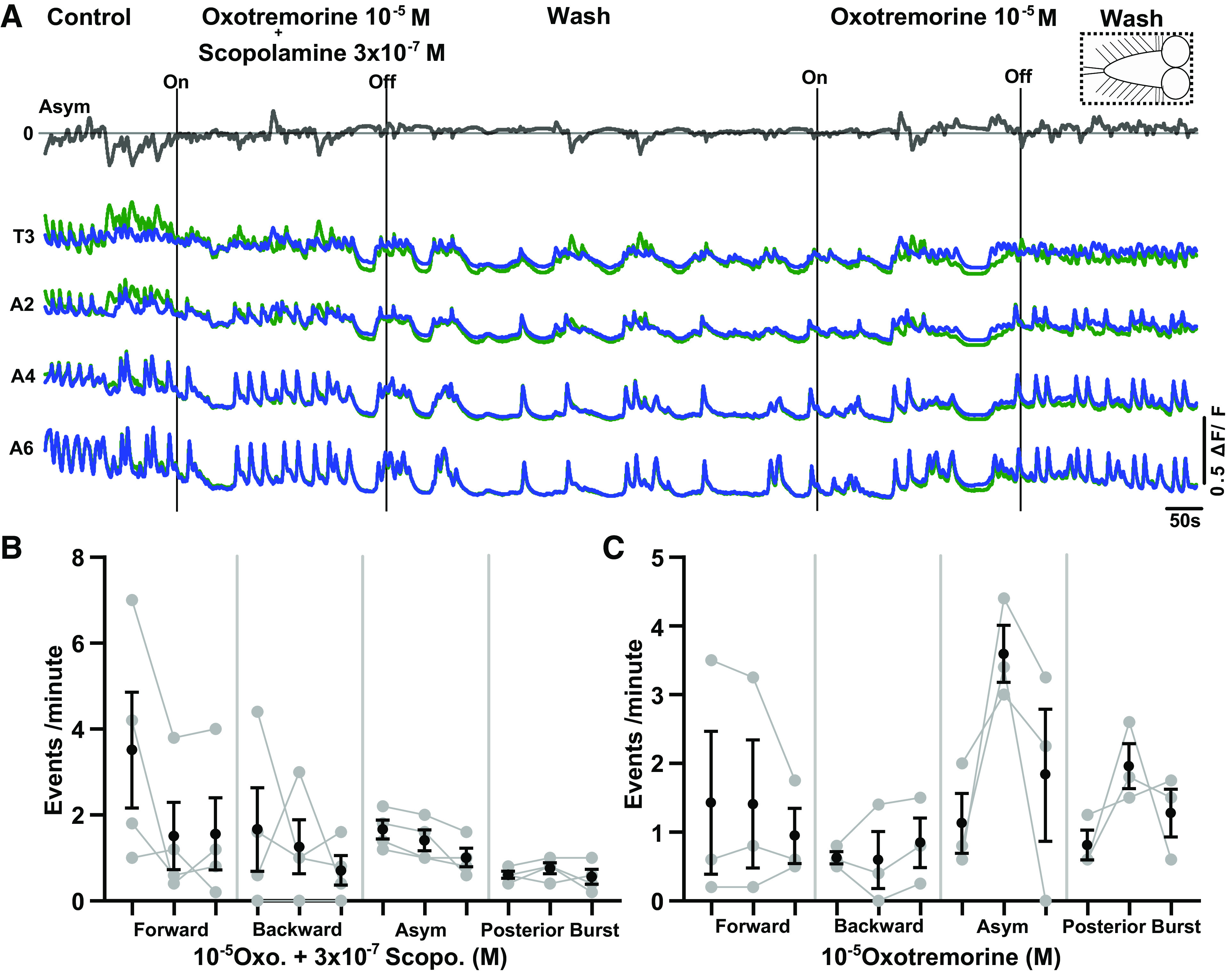
Low concentrations of scopolamine antagonize the effects of oxotremorine in the intact central nervous system (CNS). *A*: Ca^2+^ activity before during and after bath application of 3 × 10^−7^ M scopolamine and 10^−5^ M oxotremorine at same time (*left*), followed by wash (*center*) and then application of 10^−5^ M oxotremorine alone (*right*). CNS is intact (dashed box at *top right*). *B*: events per minute of different fictive motor patterns produced before, during, and after (*left* to *right*) dual application of agonist and antagonist (*n* = 4, Friedman test with Dunn’s post hoc multiple comparison; event numbers range from 0 to 74). Mean ± SE shown in black; data from each individual preparation shown in gray. *C*: pooled data showing response to oxotremorine after wash of scopolamine (*n* = 3, Friedman test with Dunn’s post hoc multiple comparison; event numbers range from 4 to 42). Same organization as in *B*.

**Figure 6.**
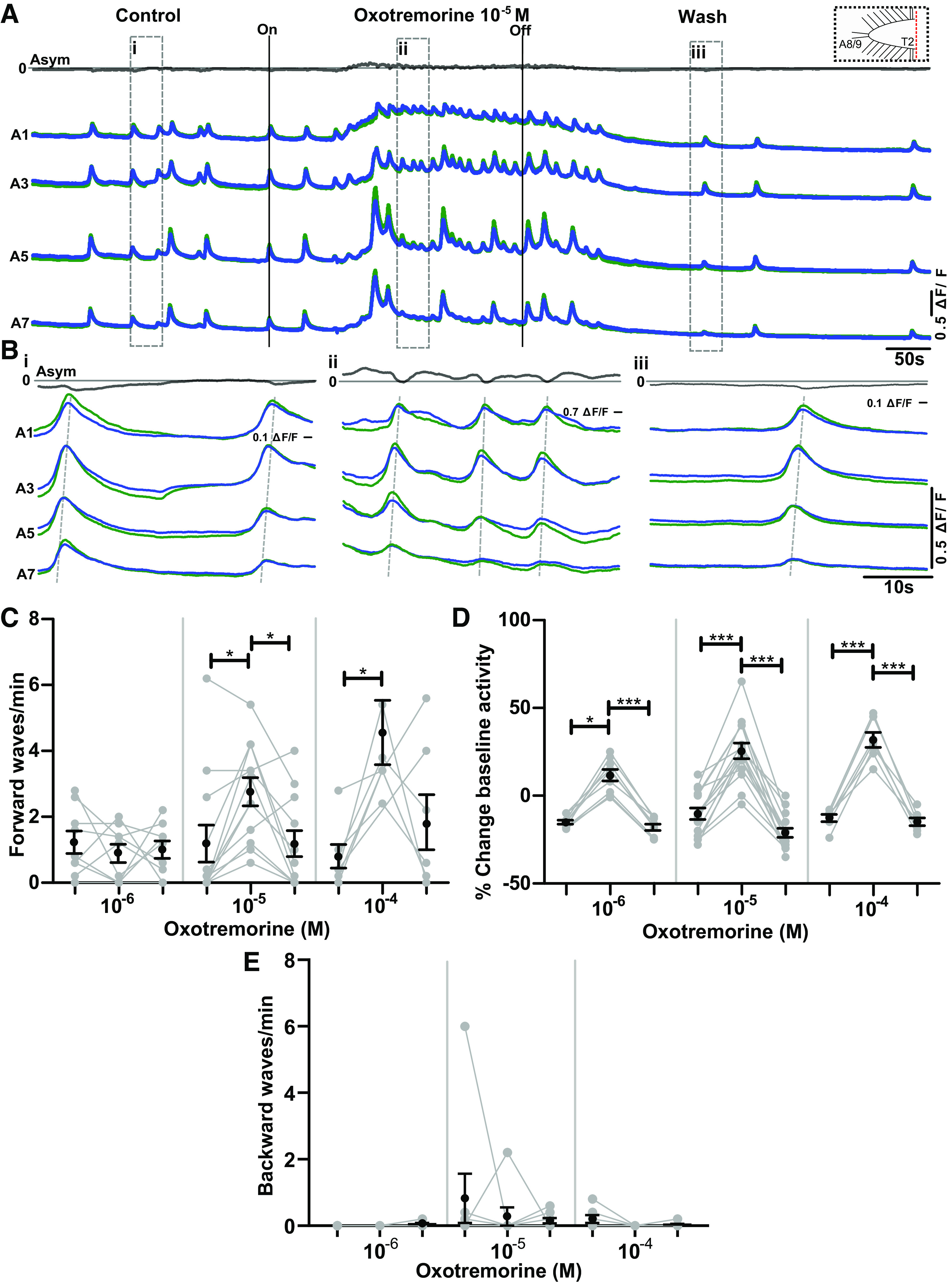
Oxotremorine increases frequency of forward waves and raises baseline Ca^2+^ levels in preparations without brain lobes. *A*: Ca^2+^ signals before, during, and after application of 10^−5^ M oxotremorine in a preparation without brain lobes (dashed box at *top right*). ΔF/F, % change in fluorescence. *B*: *i*: Expanded view of Ca^2+^ signals in control period. *ii*: Expanded view of Ca^2+^ signals in oxotremorine. *iii*: Expanded view of Ca^2+^ signals in wash period. *C*: forward waves per minute produced before, during, and after (*left* to *right*) application of different concentrations (10^−6^, 10^−5^, 10^−4^ M) of oxotremorine. Mean ± SE shown in black; data from each individual preparation shown in gray. *D*: summary of baseline fluorescence changes before, during, and after application of oxotremorine. Same organization as in *C*. *E*: backward waves per minute produced before, during, and after (*left* to *right*) application of different concentrations (10^−6^, 10^−5^, 10^−4^ M) of oxotremorine. Same organization as in *B* and *C*. Fictive headsweeps and posterior bursts were not observed in any preparations in any condition (0/28). Significant differences among groups: *P < 0.05, ***P < 0.0001; Friedman test with Dunn’s post hoc multiple comparison tests were used. Sample sizes: *C* and *D*: *n* = 9 for 10^−6^ M, *n* = 12 for 10^−5^ M, and *n* = 7 for 10^−4^ M; event numbers range from 0 to 73.

**Figure 7.**
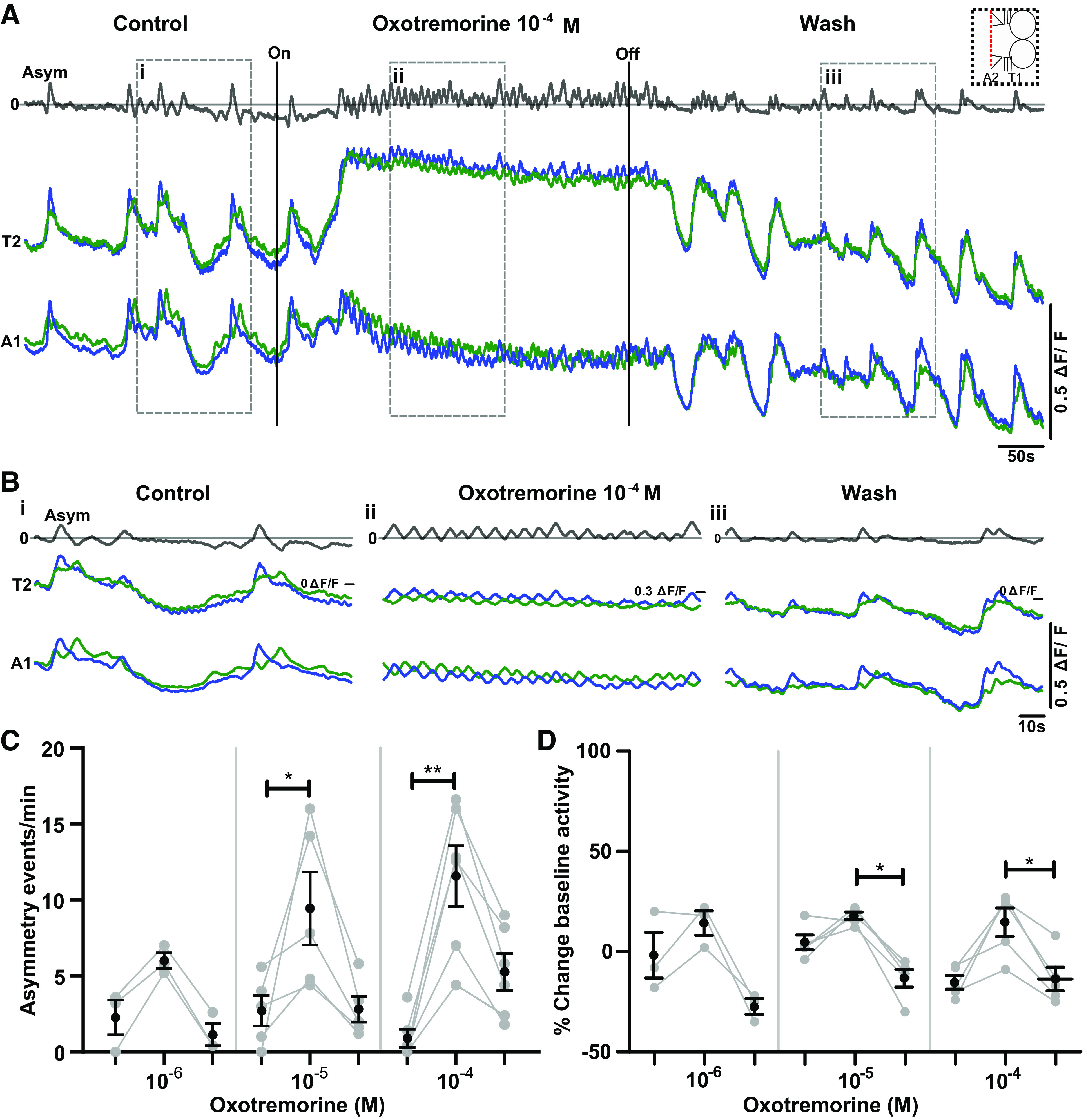
Oxotremorine increases the frequency of bilateral asymmetries and modulates baseline Ca^2+^ levels in preparations without abdominal segments. *A*: Ca^2+^ signals in thoracic and anterior abdominal segments before during and after oxotremorine in a preparation with posterior abdominal segments removed (schematic of preparation at *top right*) ΔF/F, % change in fluorescence. *B*: *i*: Expanded view of Ca^2+^ signals in control period. *ii*: Expanded view of Ca^2+^ signals during oxotremorine 10^−4^ M application. *iii*: Expanded view of Ca^2+^ signals during wash period. *C*: bilateral asymmetries per minute before, during, and after (*left* to *right*) application of 3 different concentrations of oxotremorine (10^−6^, 10^−5^, 10^−4^ M). Fictive forward and backward waves were not observed in any condition. Mean ± SE shown in black; data from each individual preparation shown in gray. *D*: summary of baseline changes in Ca^2+^ fluorescence. Same organization as in *C*. Significant differences among groups: *P < 0.05, **P < 0.005; Friedman test with Dunn’s post hoc multiple comparisons. Sample sizes: *n* = 3 for 10^−6^ M, *n* = 4 for 10^−5^ M, and *n* = 5 for 10^−4^ M; event numbers range from 28 to 130.

**Figure 8.**
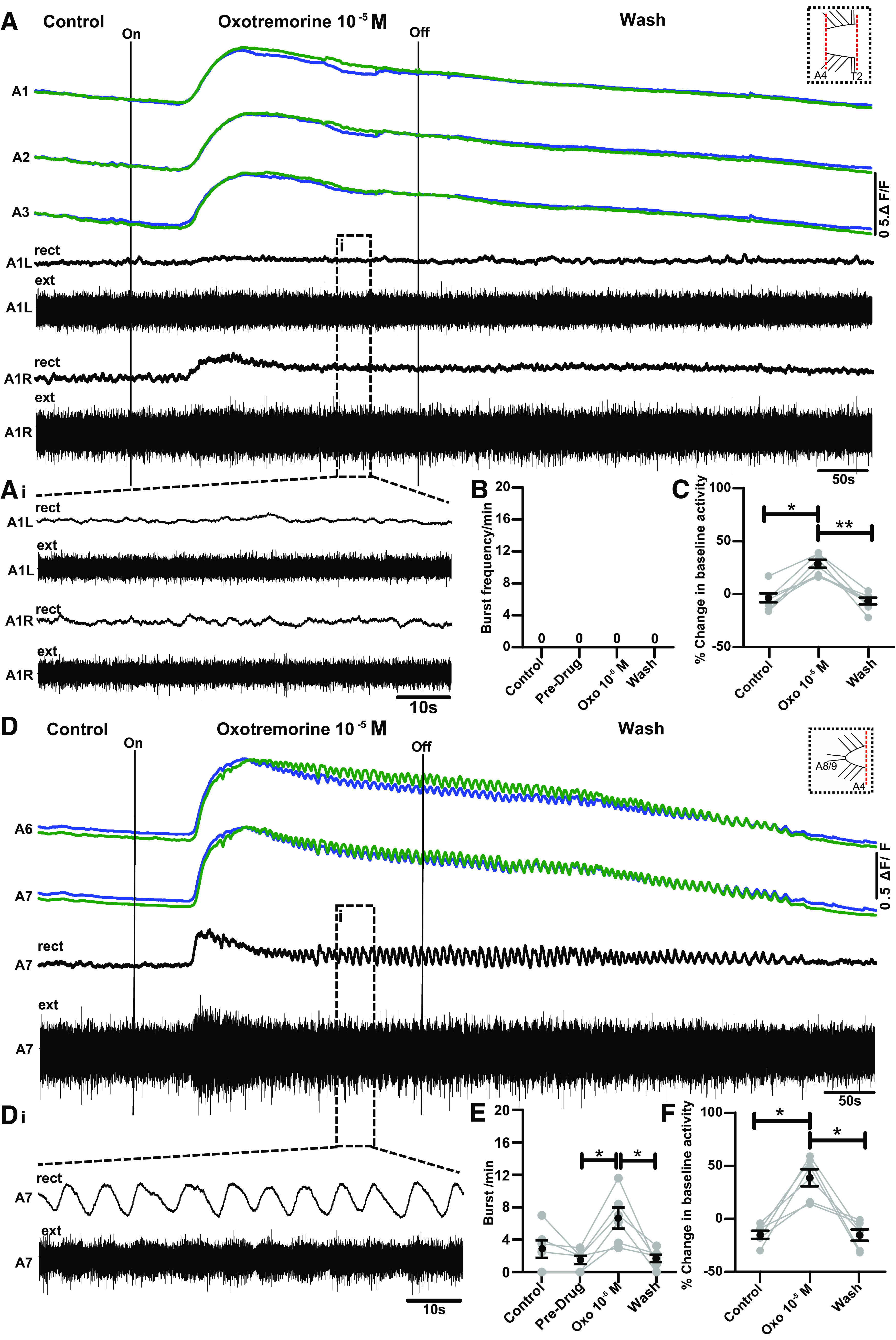
Oxotremorine triggers bursting in isolated posterior abdominal segments but not in isolated anterior abdominal segments. *A*: example of simultaneous Ca^2+^ imaging (green and blue traces) and paired nerve root recordings (black traces) from isolated anterior abdominal segments before, during, and after oxotremorine application (T2–A4, see schematic at *top right*). Extracellular nerve root signals recorded from each electrode were rectified and smoothed by filtering with a moving average filter with a time constant of 0.9 s (rect). ΔF/F, % change in fluorescence. *Ai*: expanded view of region in dashed boxes in *A*. *B* and *C*: bursts per minute and baseline fluorescence in control, “predrug” control, oxotremorine, and wash, respectively. Control period was 0–5 min after start of experiment, and predrug period followed for an additional 2 min before drug application. Control periods were segregated because of rundown of preparations. No bursts were detected in any condition (*n* = 6); however, all preparations showed reversible changes in baseline fluorescence. Mean ± SE shown in black; data from each individual preparation shown in gray. *D*: Ca^2+^ signals and single nerve root recording from posterior abdominal segments (A5–A8/9, see schematic at *top right*). *Di*: expanded view of black dashed boxes in *D*. Note that rhythmic activity is not present in *Ai*. *E* and *F*: bursts per minute and baseline fluorescence in control, control (predrug), oxotremorine, and wash, respectively (*n* = 6). Two control periods and same organization as in *B* and *C*. Significant differences among groups: *P < 0.05, **P < 0.005; Friedman test with Dunn’s post hoc multiple comparisons; event numbers range from 27 to 72.

**Figure 9.**
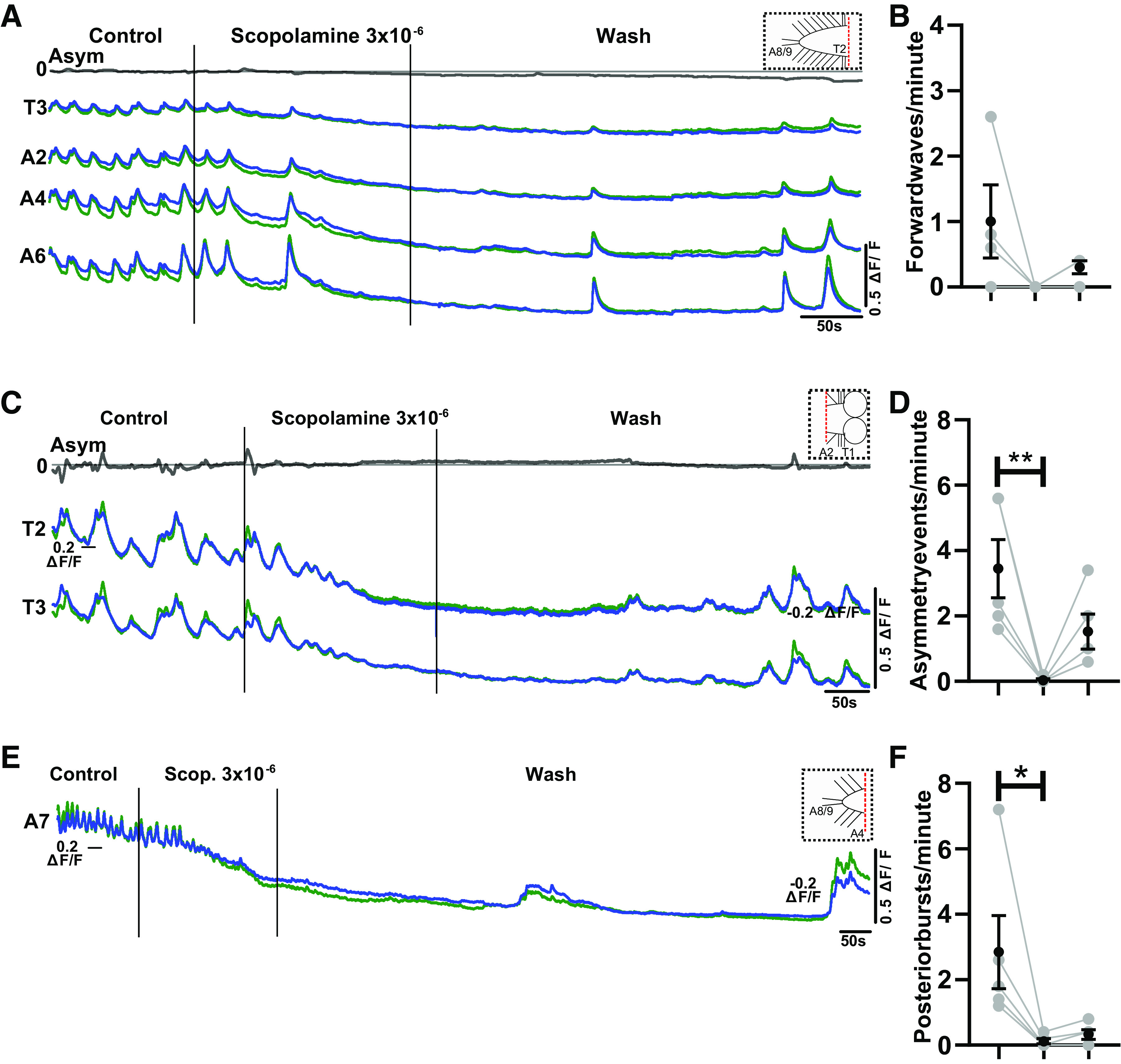
Scopolamine inhibits rhythmic activity in different ablated regions of the central nervous system (CNS). *A–F*: examples of Ca^2+^ signals from 3 different ablated parts of CNS (type of preparation depicted at *top right* in *A*, *C*, and *E*). *B*, *D*, and *F*: events per minute before, during, and after (*left* to *right*) scopolamine. Note complete suppression of rhythmic activity and partial recovery in wash. Mean ± SE shown in black; data from each individual preparation shown in gray. Sample sizes in *B*, *D*, and *F*: *n* = 4, 5, and 5, respectively; event numbers range from 0 to 46. Significant differences among groups: *P < 0.05, **P < 0.005; Friedman test with Dunn’s post hoc multiple comparisons). ΔF/F, % change in fluorescence.

The isolated CNS produced four distinct rhythmic motor patterns in physiological saline (Fig. 2), which mirror patterns of actual activity seen in intact animals (27). The four patterns were *1*) fictive forward waves (defined as bilaterally symmetric Ca^2+^ activity that started in posterior-most segments of the VNC and propagated to anterior-most segments; Fig. 2*Ci*); *2*) fictive backward waves (defined as bilaterally symmetric Ca^2+^ activity that started in anterior-most segments and propagated to posterior-most segments; *Fig. 2Cii*); *3*) bilaterally asymmetric activity, i.e., fictive headsweeps (defined as Ca^2+^ activity peaks in anterior segments on one side of the VNC and showing at least 4% difference in ΔF/F between left and right ROIs; Fig. 2*Ciii*, indicated with black dots); and *4*) posterior bursts (defined as bilaterally symmetric bursts of activity in posterior segments A8–A5 that were not followed by forward waves). Posterior bursts were termed “aborted waves” in a previous publication because they show qualitative similarities to locomotor waves that initiate but fail to propagate through medial segments (27) (Fig. 2*Ciii*, magenta dashed lines). In all contexts, we used signal processing software to automatically detect peaks and troughs in ROI data associated with different types of motor patterns. There was considerable variability in the numbers of events in analysis windows. In Figs. 3–9, the mean ± SD of raw event numbers for separate types of motor programs across all experiments was 8.37 ± 10.87, with a minimum of 0 events and a maximum of 130 events within an analysis window (*n* = 7,934 events across all experiments).

As a first step, to assess whether muscarinic signaling modulates rhythmic activity in the larval CNS, we bath applied the muscarinic acetylcholine receptor (mAChR) agonist oxotremorine and measured resulting changes in fictive motor patterns in the isolated CNS (Fig. 3, *A* and *B*). We used concentrations (10^−6^–10^−4^ M) that have been shown to activate both A-type and B-type *Drosophila* mAChRs in previous work (38, 39, 46). Bath-applied oxotremorine increased the frequency of bilaterally asymmetric events in anterior regions of the VNC (i.e., fictive headsweeps) in a dose-dependent manner (*P* < 0.0001 in 10^−4^ M, *P* < 0.05 in 10^−5^ M; Fig. 3*E*). The two highest concentrations (10^−5^ and 10^−4^ M) also significantly increased the frequency of bilaterally symmetric bursts in posterior regions (i.e., aborted forward waves) (*P* < 0.05 in both 10^−4^ and 10^−5^ M; Fig. 3*F*). Forward wave frequencies were also reduced significantly at the highest concentration of oxotremorine (*P* < 0.005) (Fig. 3*C*; 10^−4^ M). Backward wave frequencies were not significantly affected at any concentrations of oxotremorine (Fig. 3*D*). Forward waves and backward waves in the isolated CNS underlie forward and backward locomotion in intact animals. After drug application, most preparations returned to activity levels similar to those seen in control conditions (note gray scatterplots in Fig. 3). These intact CNS preparations spontaneously produced a variety of fictive behaviors in control conditions; as a result, it was difficult to ascertain with certainty whether oxotremorine induced any given motor pattern within local circuits of the VNC.

To test whether muscarinic signaling is necessary for rhythm generation, we bath applied the nonselective mAChR antagonist scopolamine to the isolated intact CNS and measured resulting changes in activity patterns (Fig. 4, *A* and *B*). We observed significant decreases in forward waves, backward waves, and fictive headsweep frequencies in 3 × 10^−4^ M scopolamine (*P* < 0.05, *P* < 0.05, *P* < 0.001, respectively; Fig. 4, *C–E*) with little to no recovery in the wash periods. There was no significant decrease in forward and backward wave frequencies at lower scopolamine concentrations; however, scopolamine decreased the frequency of bilateral asymmetries at 3 × 10^−6^ M (*P* < 0.005) and showed a similar, albeit nonsignificant, trend at 3 × 10^−5^ M (*P* > 0.05). Scopolamine had no effect on posterior burst frequency at any concentration (*P* > 0.05) (Fig. 4*F*).

In the next set of experiments, we wanted to determine whether the effects of oxotremorine could be antagonized by the addition of scopolamine (Fig. 5). We bath applied a dose of scopolamine (3 × 10^−7^ M) that had minimal effects on the isolated CNS together with a concentration of oxotremorine (10^−5^ M) that evoked significant effects on motor output (Fig. 3). The increase in event frequencies (i.e., fictive headsweeps and posterior bursts) normally observed in oxotremorine was blocked in the presence of scopolamine (Fig. 5*B*, *n* = 4). To confirm that these preparations were actually responsive to oxotremorine in the absence of scopolamine, oxotremorine was applied alone after both drugs were washed out. Although the overall effects were not significant, all preparations showed increases in the frequency of fictive headsweeps and posterior bursts during this second oxotremorine application (Fig. 5*C*), suggesting a trend toward reversibility.

After confirming that manipulation of mAChR signaling was an effective means to induce or suppress rhythm generation in different regions, we then examined the effects of modulating mAChR signaling on isolated regions. Previous work has shown that the larval VNC is able to produce rhythmic motor patterns without brain lobes (27). To determine how signaling through mAChRs modulates activity generated intrinsically in the VNC, we surgically removed brain lobes, leaving the subesophageal zone (SEZ), thorax, and abdomen intact, and then recorded activity before, during, and after bath application of oxotremorine (Fig. 6*A*). We confirmed that motor neurons were indeed active in a subset of preparations with nerve root recordings and that hemisegmental Ca^2+^ signals were representative as in intact CNS preparations (Supplemental Fig. S1; see https://doi.org/10.6084/m9.figshare.17118632). We then used Ca^2+^ imaging data for all subsequent analyses (Fig. 6, *A–D*). When the brain lobes were removed, preparations became biased toward fictive forward waves. Backward waves were only present in a subset of preparations; frequency of backward waves did not change significantly in any concentration of oxotremorine (Fig. 6*E*). Fictive headsweeps and posterior bursts were not observed in any preparations in any conditions (0/28). Brain lobe-ablated preparations did, however, show a significant increase in fictive forward wave frequencies upon application of 10^−4^ M and 10^−5^ M oxotremorine (*P* < 0.05) (Fig. 6*C*). In these preparations, we also observed a reversible increase in overall baseline fluorescence in 10^−4^ M and 10^−5^ M oxotremorine (*P* < 0.001 and *P* < 0.05, respectively; Fig. 6*D*). In brain lobe-excised preparations, the effects of 10^−5^ M oxotremorine (Fig. 6) were antagonized with low concentrations of scopolamine (3 × 10^−7^ M), as observed in intact CNS preparations (Supplemental Fig. S2; see https://doi.org/10.6084/m9.figshare.17118644).

Next we set out to determine how mAChR modulates motor patterns produced in preparations without abdominal segments. In these experiments, we ablated the abdominal segments of the ventral nerve cord and recorded Ca^2+^ activity in remaining hemisegments. In these preparations, no obvious wavelike activity was observed, but a subset of preparations did show periods of bilaterally synchronized bursts of activity (Fig. 7, *A* and *B*). The frequency of fictive headsweeps increased in a dose-dependent manner in oxotremorine (10^−4^ M: *P* < 0.01, 10^−5^ M: *P* < 0.05, 10^−6^ M: nonsignificant but trending toward increase; Fig. 7*C*). A nonsignificant trend toward an increase in baseline fluorescence was also observed at all concentrations of oxotremorine (Fig. 7*D*). This was followed by decreases in baseline fluorescence in the wash period (10^−4^ M: *P* < 0.05, 10^−5^ M: *P* < 0.05; Fig. 7*D*). Motor nerve root recordings in these types of preparations showed similar increases in motor activity in oxotremorine, suggesting that our Ca^2+^ imaging did indeed reflect changes in actual motor output (Supplemental Fig. S3; see https://doi.org/10.6084/m9.figshare.17118647).

To further examine mAChR-mediated effects on different parts of the VNC, we ablated brain lobes and all segments anterior to thoracic segment T2 while also transecting the posterior abdomen at abdominal segment A4 (Fig. 1*D*). We were then able to simultaneously record activity from both anterior and posterior segments of the VNC in isolation from each other (Fig. 8). Isolated anterior abdominal segments showed a reversible increase in baseline fluorescence (*P* < 0.01; Fig. 8, *A* and *C*) but did not produce any rhythmic activity (Fig. 8, *A* and *B*, and Supplemental Fig. S4; see https://doi.org/10.6084/m9.figshare.17118650). In contrast, isolated posterior segments initially showed robust rhythmic activity in an initial control period but then often slowed or stopped after 5–7 min of recording (Fig. 8, *D*–*F*). After ∼5 min, bursting activity was measured for an additional 2 min during a predrug control period. Compared with the predrug periods, all preparations showed reversible changes in baseline fluorescence as well as reversible increases in rhythmic activity in oxotremorine (Fig. 8, *D*–*F*, *P* < 0.05). Closer examination of the rhythmic Ca^2+^ activity produced by posterior segments in oxotremorine revealed that Ca^2+^ signals were largely synchronous across segments (Supplemental Fig. S5; see https://doi.org/10.6084/m9.figshare.17118659).

Next, we aimed to investigate whether scopolamine could inhibit motor pattern generation in transected parts of the VNC (Fig. 1*D*). In three types of transected preparations (brain lobes removed, posterior abdomen removed, all anterior segments removed), application of low doses of scopolamine (3 × 10^−6^ M) inhibited or abolished rhythmic activity (*P* > 0.05, *P* < 0.005, *P* < 0.05, respectively; Fig. 9). All preparations showed at least partial recovery of rhythmicity in wash periods, confirming that rhythm-generating circuits retained functionality after scopolamine application.

**Figure 10.**
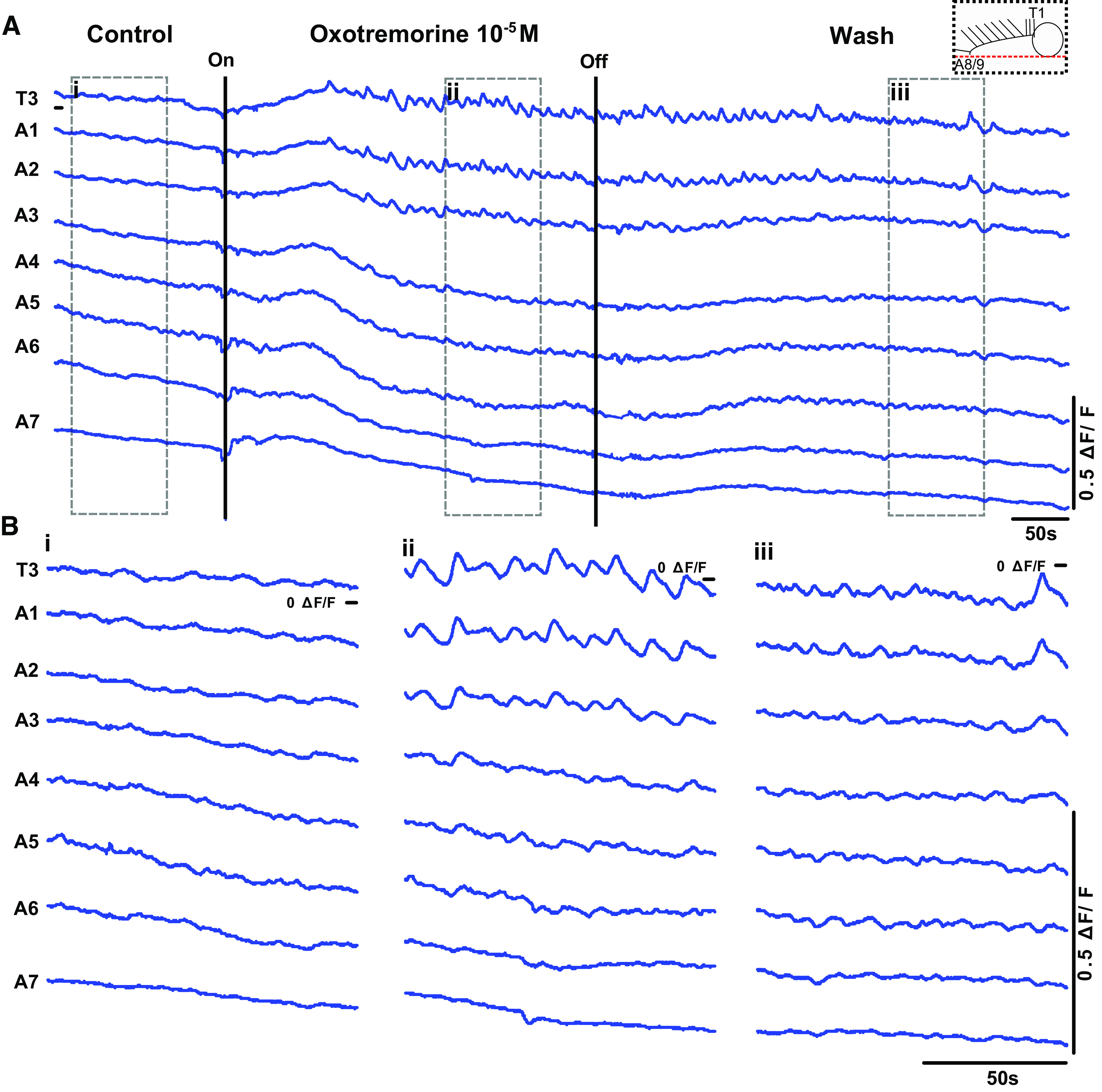
Oxotremorine triggers bursting in anterior regions in longitudinally hemisected preparations. *A*: representative traces of Ca^2+^ signals from a longitudinally hemisected preparation before, during, and after oxotremorine application (schematic of the preparation shown at *top right*). *B*: expanded view of Ca^2+^ signals in control period (*i*), 10^−5^ M oxotremorine (*ii*), and wash period (*iii*). Note bursts of activity in anterior regions. Results representative of 3 preparations. ΔF/F, % change in fluorescence.

Finally, to determine whether midline connections are required to produce rhythmic activity, we ablated one entire side of the CNS (Fig. 1*D*) and measured responses to oxotremorine (Fig. 10, *A* and *B*). Bath application of oxotremorine to these preparations triggered an increase in baseline fluorescence and largely synchronous rhythmic bursting in anterior regions but did not induce fictive forward or backward waves (Fig. 10*A*, *n* = 3).

Overall, our results suggest the presence of multiple rhythm-generating modules in the larval VNC that are strongly modulated by mAChR signaling, with distinct modules present in anterior brain and thoracic regions as well as in posterior abdominal segments. Activation of mAChRs promotes bursting at distal ends of the larval VNC but does not promote wavelike activity, except in preparations without brain lobes. Finally, midline connections do not appear to be strictly required for muscarinic-dependent rhythm generation in anterior regions.

## DISCUSSION

Here we used a combination of electrophysiology, Ca^2+^ imaging, and pharmacology in isolated CNS preparations to explore the architecture of rhythm generation in the *Drosophila* larval locomotor system. We pharmacologically manipulated mAChR signaling and measured the resulting effects on a variety of rhythmic motor patterns including forward and backward waves, fictive headsweeps, and posterior bursts. In intact CNS preparations, the mAChR agonist oxotremorine did not strongly affect the frequency of forward or backward fictive locomotion but did potentiate fictive headsweeps and bursting in posterior abdominal regions. The mAChR antagonist scopolamine blocked the effects of oxotremorine and inhibited rhythmic activity when applied alone. In reduced preparations, oxotremorine potentiated rhythm generation in isolated distal regions of the VNC, whereas scopolamine abolished rhythmic activity in the same types of preparations. Overall, these results suggest that mAChR signaling plays a role in generating and modulating rhythmic activity within the larval CNS and provide initial evidence for the presence of at least two distinct mAChR-dependent rhythm-generating modules: one located in brain and thoracic segments that generates bilaterally asymmetric activity and one located in posterior-most abdominal segments that generates bilaterally symmetric activity that, in turn, initiates forward waves.

To manipulate muscarinic signaling, we used a pharmacological approach in which we bath applied different concentrations of the mAChR agonist oxotremorine (10^−4^ to 10^−6^ M) and the mAChR antagonist scopolamine (3 × 10^−4^ to 3 × 10^−7^ M). We chose these concentrations because they have been shown to activate *Drosophila* mAChRs [and not nicotinic acetylcholine receptors (nAChRs)] in heterologous expression systems (38, 39) and because they are equivalent to or lower than those used in other studies of vertebrate (47, 48) and arthropod (14, 34, 35, 37, 46, 49) locomotor networks. Similarly, the concentrations of antagonists used are known to block *Drosophila* mAChRs and, at the lowest concentration, are primarily selective for A- and C-type receptors while not strongly affecting B-type receptors (38, 39). Critically, the effects of oxotremorine could be fully antagonized by coapplication of low (i.e., <10^−6^ M) doses of scopolamine, suggesting that oxotremorine is indeed acting through mAChR receptors and not through off-target mechanisms.

Taking a pharmacological approach in an in vitro preparation limited our ability to precisely target specific receptor subtypes (drugs specifically targeting *Drosophila* subtypes are not available). Although it is possible to use RNA interference (RNAi) to suppress expression of specific mAChR subtypes (40, 50), these genetic approaches manipulate mAChR expression over developmental timescales. This raises the possibility that any phenotypes observed in RNAi studies are the end result of developmental compensation in response to misexpression of receptors. A major advantage of our pharmacological approach is that it allowed us to acutely manipulate mAChR signaling on a timescale of minutes, thereby providing a clear view of receptor function without the confounding variable of developmental compensation.

Previous work in *Drosophila* larvae has shown that longer-term pharmacological and genetic manipulations of mAChR activity can lead to nonintuitive changes in larval behaviors. Feeding animals muscarine for 24 h resulted in lower forward peristaltic wave frequency, whereas feeding them antagonists had a similar effect (40). In another study, larvae fed the mAChR antagonist atropine showed relatively normal locomotion but failed to navigate away from noxious odors (50). Interestingly, genetic knockdown of specific subtypes of mAChR with RNAi gives a different set of results. Suppressing expression of A- and C-type receptors in a variety of cell populations actually leads to an increase in forward wave frequency (40). One explanation for the mismatch in behavior effects between pharmacological and genetic manipulation observed in Ref. 40 could be that both manipulations are occurring over relatively long periods (1–4 days). On this timescale, compensatory homeostatic mechanisms could be engaged (or overengaged) in response to the manipulations, thereby complicating interpretation of resulting behaviors.

In previous work, acute application of oxotremorine and other muscarinic agonists triggered peristaltic waves of activity in semi-intact larval preparations and potentiated sensori-motor circuitry; conversely, mAChR antagonists typically suppressed activity (40, 46). In these studies, motor activity was measured by recording excitatory junctional potentials (EJPs) at the neuromuscular junction and sensory feedback was intact in semi-intact preparations. Recent work has also examined activity in single nerve roots in isolated CNS preparations in response to mAChR agonists and antagonists (47). This previous body of work largely agrees with our findings, namely that muscarinic agonists tend to potentiate rhythmic activity within the larval CNS and antagonists suppress activity. However, there are notable exceptions to these trends in adult *Drosophila*, where signaling through mAChRs leads to inhibitory effects in certain neurons (51). Although insightful, previous electrophysiological studies in larvae provide limited views of the overall activity of the larval locomotor network because they only recorded activity in one or two body segments at a time.

Our imaging experiments allowed us to measure rhythmic activity across most VNC segments through a combination of Ca^2+^ imaging and simultaneous extracellular nerve root recordings. Although this approach did not allow us to distinguish between identified cell types, it did provide an overview of all activity in the VNC. Importantly, we confirmed in multiple types of preparations that measuring Ca^2+^ signals in defined regions within hemisegments is reflective of motor output from a given hemisegment. First, we wanted to test how bath application of the agonist oxotremorine would affect spontaneous rhythmic activities produced by the isolated larval CNS. The recordings were at first difficult to interpret, as the effects on forward or backward wave frequency were inhibitory at high agonist concentrations while also clearly increasing activity overall in the network (Fig. 3). Further analysis revealed that the main effect of oxotremorine was actually to potentiate bilateral asymmetries (fictive headsweeps) in thoracic regions and potentiate posterior bursting in posterior abdominal regions. As activation of fictive headsweep rhythms increased with increasing oxotremorine concentration, this appeared to progressively impair the VNC’s ability to generate forward waves that propagate through the entire VNC, despite showing a concomitant increase in forward wave initiation events (posterior bursts). Bath application of scopolamine suppressed generation of multiple fictive motor programs, with especially strong effects on fictive headsweeps. One counterintuitive result was that in the intact CNS both agonist and antagonist inhibited generation of wavelike activity. This result makes sense in a model where oxotremorine activates the headsweep module, which in turn disrupts wave propagation. Overall, our intact CNS data suggest that mAChR signaling enables the generation of rhythmic motor patterns in the larval VNC that require intersegmental and/or bilateral coordination. These results are similar to those obtained in the locust flight CPG (35) and various insect CPGs (34–37) including *Manduca* larval crawl CPG (14). In vertebrate spinal cord, signaling through NMDA receptors appears to play a key role in raising cytosolic Ca^2+^, which then contributes to initiation of rhythmic activity in both intact and hemisected preparations (2, 5, 52). Signaling through mAChRs may be playing an analogous role in promoting rhythmogenesis in insect locomotor CPGs, with activation of A- and/or C-type mAChRs leading to release of intracellular Ca^2+^ stores through phospholipase C-triggered transduction pathways (39).

We noted in our initial experiments that the initiation of vigorous bouts of fictive headsweeps and posterior bursting appeared to suppress a preparation’s ability to generate wavelike activity. We reasoned that oxotremorine may be triggering two competing independent rhythm generators at either end of the network. To test this idea, we performed a series of ablation experiments aimed at localizing muscarinic-dependent rhythm-generating modules. In brain lobe-ablated preparations, bilateral asymmetries were abolished and the network was biased toward generating forward waves. In these preparations, oxotremorine triggered posterior bursting that, in turn, triggered forward waves on every cycle. These effects were completely blocked by the addition of scopolamine and reversible in the same preparations after a wash period (Supplemental Fig. S2). This suggests that a local muscarinic-dependent rhythm-generating mechanism exists in abdominal regions and that this mechanism triggers forward waves. These results also provide indirect evidence of a dominance hierarchy among two rhythm generators. The presence of fictive headsweep circuitry suppresses the generation of full forward waves in agonist; however, in the absence of any fictive headsweep circuit activity in brain lobe-ablated preparations, forward waves dominate in agonist.

In the next set of experiments, we ablated abdominal regions and measured activity in preparations with just brain lobes and thoracic regions remaining. Application of oxotremorine triggered dose-dependent increases in the frequency of bilateral asymmetries; however, even in the absence of agonist, preparations generated bilaterally asymmetric activity. These results suggest that a separate rhythm-generating module in anterior regions underlies fictive headsweeps. Interestingly, recent work has independently suggested that continual lateral oscillations of anterior regions during navigation are driven by neuronal oscillators, which could be subject to neuromodulation (53). The results presented here corroborate this idea and provide an entry point to identifying the circuit components underlying headsweep oscillations.

We continued systematically removing parts of the VNC to determine how different parts of the VNC responded to oxotremorine. To confirm the presence or absence of any possible type of rhythmic activity, wherever possible, we performed both panneuronal imaging and extracellular nerve root recordings simultaneously. One striking result was that middle sections of the VNC (A1–A5) did not produce any rhythmic activity, even in oxotremorine (Fig. 8*A*). They did, however, respond to oxotremorine with a raised baseline Ca^2+^ signal that reversed in wash conditions. This last provided some limited confirmation of viability in these preparations, but it was difficult to gauge the overall health of rhythm-generating circuits in these dual-cut experiments. The absence of rhythmic activity in these preparations does not conclusively prove that those segments are incapable of generating rhythmic activity. Conversely, isolated posterior segments from the same preparations were highly rhythmogenic and showed regular bursting in control conditions and in oxotremorine (Fig. 8*D*). Bursting in these preparations appeared largely synchronous across segmental boundaries and the midline; however, wavelike activity may have been masked by the fact that we were imaging panneuronally. This could be resolved in future studies by driving the expression of fast GCaMP constructs in motor neurons alone. One possible explanation for the difference in rhythmogenicity seen in isolated anterior and posterior abdominal regions is that oscillators are present in each segment but show varying levels of rhythmogenicity. This is somewhat at odds with existing models of the larval locomotor system that assume that each hemisegment contains an identical oscillator that is then coupled to other oscillators in every adjacent hemisegment (32). Our work suggests that the larval VNC does contain strongly rhythmogenic modules in anterior-most (thoracic/brain) and posterior-most (A6–A8) segments. Medial segments may or may not be intrinsically less rhythmogenic. Importantly, our results do not definitively rule out the presence of oscillators in medial hemisegments. Oscillators may be present but simply less rhythmogenic or indeed damaged by our ablations. Oscillators could also be present but conditionally activated by other modulatory mechanisms. In our previous work we noted that preparations consisting of brain lobes, thorax, and anterior abdomen could sometimes initiate bouts of both forward and backward waves (27); this observation, coupled with our results here, argues for the presence of conditional or “latent” oscillators in medial segments that lie toward the bottom of a dominance hierarchy of CPG modules. These weaker oscillators would tend to be entrained by, and follow, stronger oscillators in distal segments, but in the absence of this entrainment the latent oscillators could “rise up” in the hierarchy and initiate rhythmic wavelike activity. These types of conditional oscillators could conceivably appear as effectively “silent” in our double-ablation experiments. Further work aimed at probing the rhythmogenic abilities of small numbers of hemisegments under different modulatory conditions could help resolve this issue. This conceptual model would be partly in line with previous work in vertebrates showing the presence of rostral-caudal gradients of rhythmogenicity and resulting patterns of entrainment in spinal circuitry (2, 54–56). Notably, however, in vertebrates these gradients are unimodal, with rhythmogenicity steadily declining rostral to caudal. In contrast, in the soft-bodied leech all segments appear equally capable of producing crawling motor programs in isolation in the presence of dopamine, with both descending neurons and local circuits providing substrates for intersegmental coordination (15).

In the intact CNS, the muscarinic antagonist scopolamine reduced the frequency of fictive motor patterns but did not completely abolish rhythmic activity (Fig. 4). In contrast, relatively low doses of scopolamine completely abolished endogenous rhythmic activity in brain-ablated, isolated anterior, and isolated posterior segments (Fig. 9). These data suggest that muscarinic signaling is indeed critically important for enabling local rhythm generation in distal rhythm generators.

Our work complements previous work showing that pharmacological block of nicotinic acetylcholine receptors (nAChRs) abolishes all rhythmic synaptic activity (57) and calcium oscillations (J. Booth, Pulver laboratory, personal communication), suggesting that fast excitation generally, and specifically signaling through nAChRs, is required for rhythm generation. Conversely, bath application of picrotoxin leads to seizurelike activity followed by eventual collapse of rhythmic activity (58), suggesting that inhibition generally, and specifically inhibition mediated by GABAA and/or glutamate-gated chloride channels, is also critical for rhythm generation in this system. Seizurelike activity patterns in disinhibited networks show qualitative similarities to some of the activity patterns that we observed in oxotremorine (see especially Fig. 5). This raises the possibility that, as in spinal networks, maintaining appropriate balances of excitation and inhibition in the larval VNC is critical to maintaining activity patterns within physiological ranges. Excitatory and inhibitory interneurons with critical roles in wave pattern generation have recently been identified in the larval CNS (21, 23, 33, 59–61); however, further signaling requirements for rhythm generation in this system remain unclear. In particular, the contribution of rhythmogenic ionic currents (17, 62) is largely unexplored and could be the focus of future work.

In a final set of experiments, we asked whether bilaterally asymmetric motor patterns in thoracic regions require connections across the midline. We longitudinally transected the entire CNS and recorded endogenous and oxotremorine-evoked activity. These preparations were not able to produce wavelike activity or posterior bursting; however, they were able to produce synchronous activity in thoracic segments in the presence of oxotremorine (Fig. 10). These results suggest that bilateral asymmetric activity arises from interactions between independent rhythm-generating modules located on either side of the CNS, as is the case in vertebrate spinal cord (2). These results also tentatively suggest that contralateral connections may be required for rhythm generation in posterior segments. Importantly, the lack of rhythmic activity in posterior regions in hemisected preparations should not be regarded as conclusive evidence for critical contralateral connections, especially given the difficulty of making precise longitudinal cuts at the narrow posterior tip of the VNC.

Overall, this work identifies mAChRs as important modulators of rhythm generation in the *Drosophila* larval CNS. We provide evidence for presence of distinct mAChR-dependent rhythm-generating modules in distal regions of the larval VNC and initial evidence for the presence of dominance hierarchies among these modules. Previous work suggests that mAChRs are widely distributed in the brain and VNC of larvae (50); however, it is unclear exactly how mAChRs are distributed among identified interneurons. Future work aimed at carefully characterizing the spatial distribution of mAChRs among identified cell types in the larval VNC could be a productive next step toward identifying the interneuron cell types at the core of rhythm-generating circuits in the larval VNC.

## DATA AVAILABILITY

All data underlying figures and genetic constructs are available upon request to the corresponding author.

## SUPPLEMENTAL DATA

10.6084/m9.figshare.17118632 Supplemental Fig. S1: https://doi.org/10.6084/m9.figshare.17118632.

10.6084/m9.figshare.17118644 Supplemental Fig. S2: https://doi.org/10.6084/m9.figshare.17118644.

10.6084/m9.figshare.17118647 Supplemental Fig. S3: https://doi.org/10.6084/m9.figshare.17118647.

10.6084/m9.figshare.17118650 Supplemental Fig. S4: https://doi.org/10.6084/m9.figshare.17118650.

10.6084/m9.figshare.17118659 Supplemental Fig. S5: https://doi.org/10.6084/m9.figshare.17118659.

## GRANTS

This work was supported by the Wellcome Trust through an ISSF award (105621/Z/14/Z) to the University of St Andrews. It was also supported by a Biotechnology and Biological Sciences Research Council (BBSRC) project grant (BB/M021793/1) awarded to S.R.P., a BBSRC CASE studentship awarded to J.M. (BB/M010996/1), and a donation from Kaunas Industrial Water Supply (Kauno Pramoninis Vandentiekis, Kaunas, Lithuania) in support of J.J.

## DISCLOSURES

No conflicts of interest, financial or otherwise, are declared by the authors.

## AUTHOR CONTRIBUTIONS

J.J. and S.R.P. conceived and designed research; J.J. and J.M. performed experiments; J.J. analyzed data; J.J., J.M., and S.R.P. interpreted results of experiments; J.J. prepared figures; J.J. and S.R.P. drafted manuscript; J.J., J.M., and S.R.P. edited and revised manuscript; J.J., J.M., and S.R.P. approved final version of manuscript.
